# Supplementary figures and images for: Crosstalk between Mesenchymal Stem Cells and Cancer Stem Cells Reveals a Novel Stemness-Related Signature to Predict Prognosis and Immunotherapy Responses for Bladder Cancer Patients
Source: Int J Mol Sci. 2023 Mar 1;24(5):4760. doi: 10.3390/ijms24054760 (PMC10003512; doi:10.3390/ijms24054760)

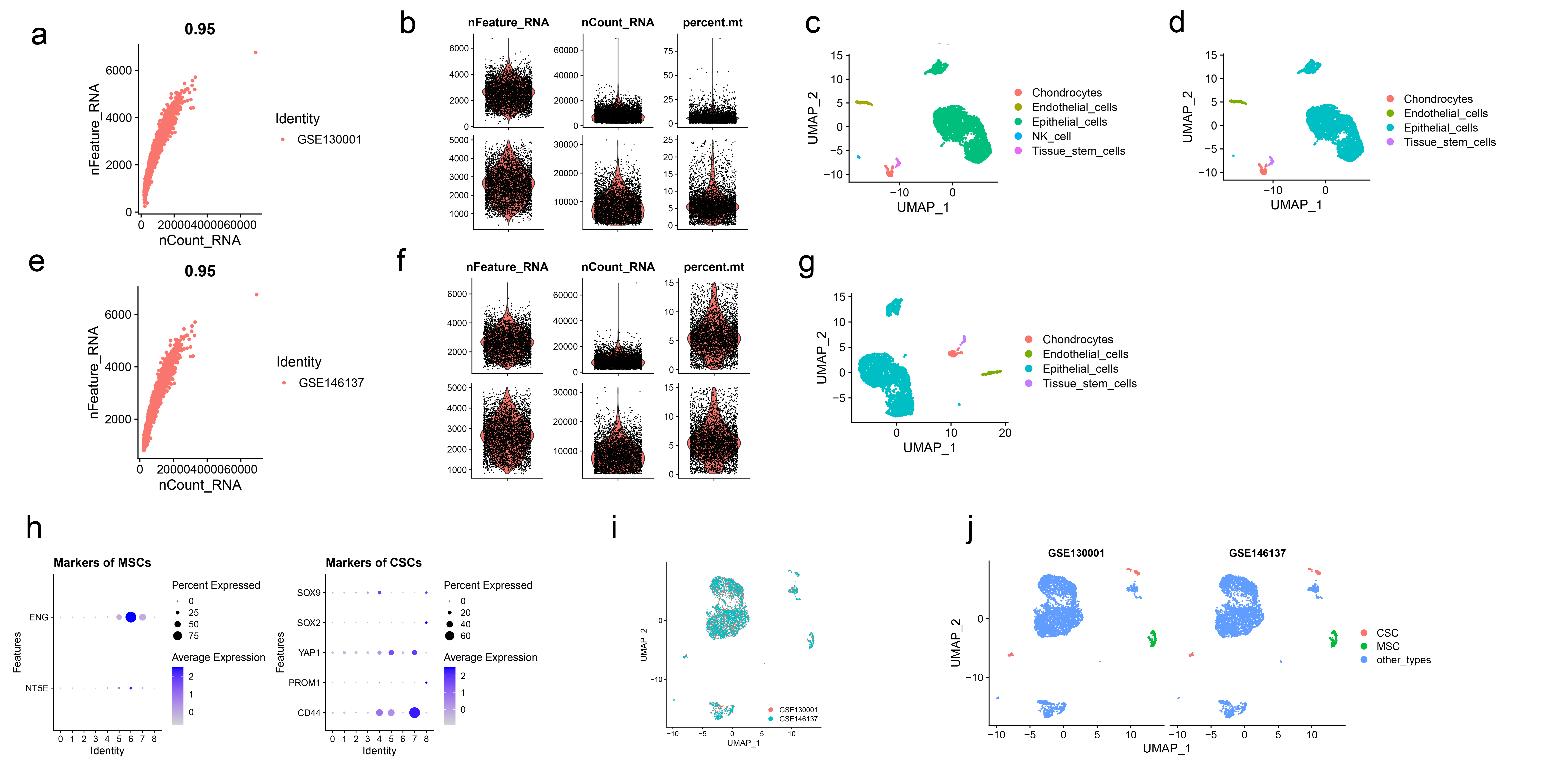

Supplement: Supplementary file 1 [file ijms-24-04760-s001.zip › Figure S1.tif]

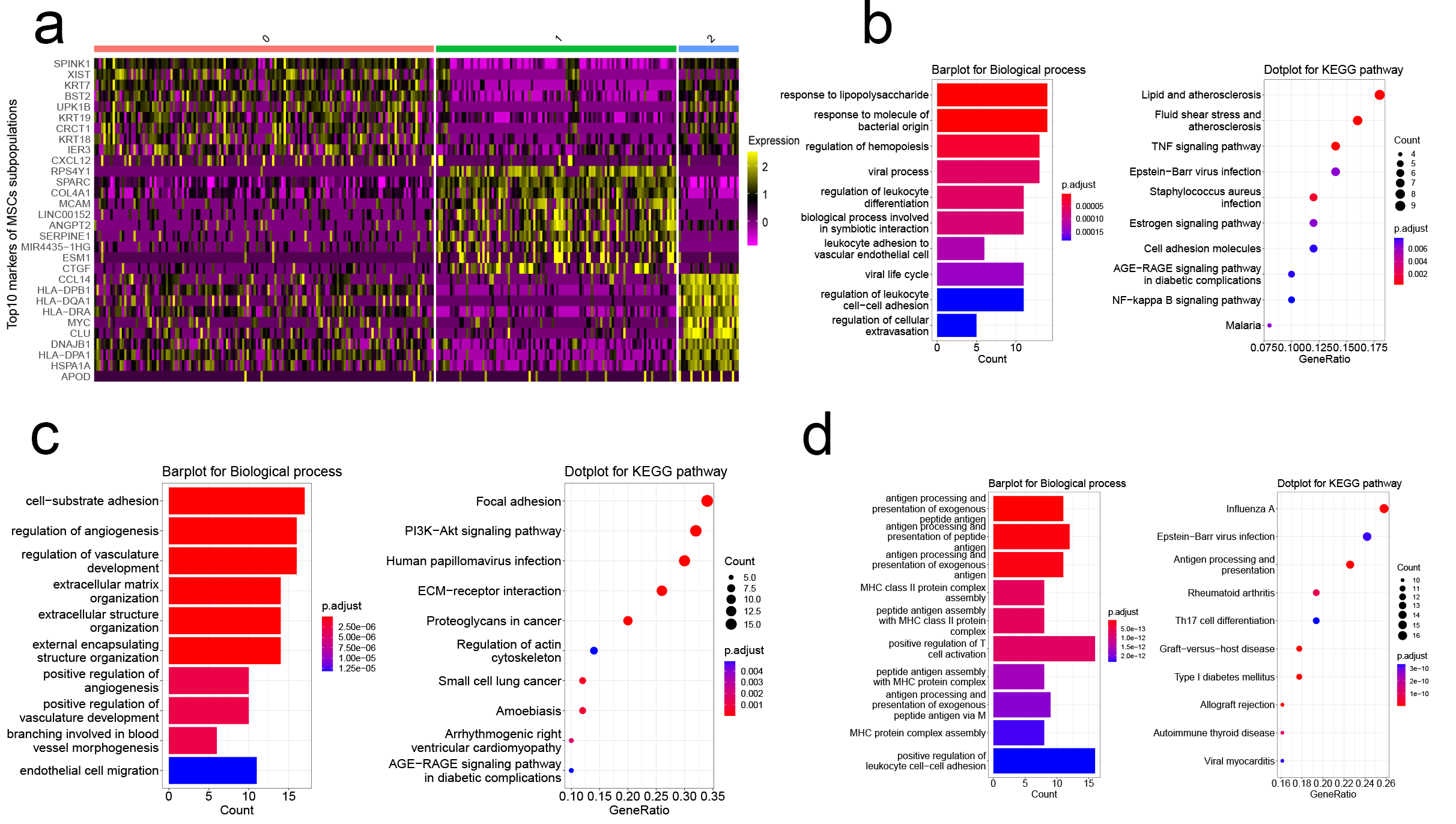

Supplement: Supplementary file 1 [file ijms-24-04760-s001.zip › Figure S2.tif]

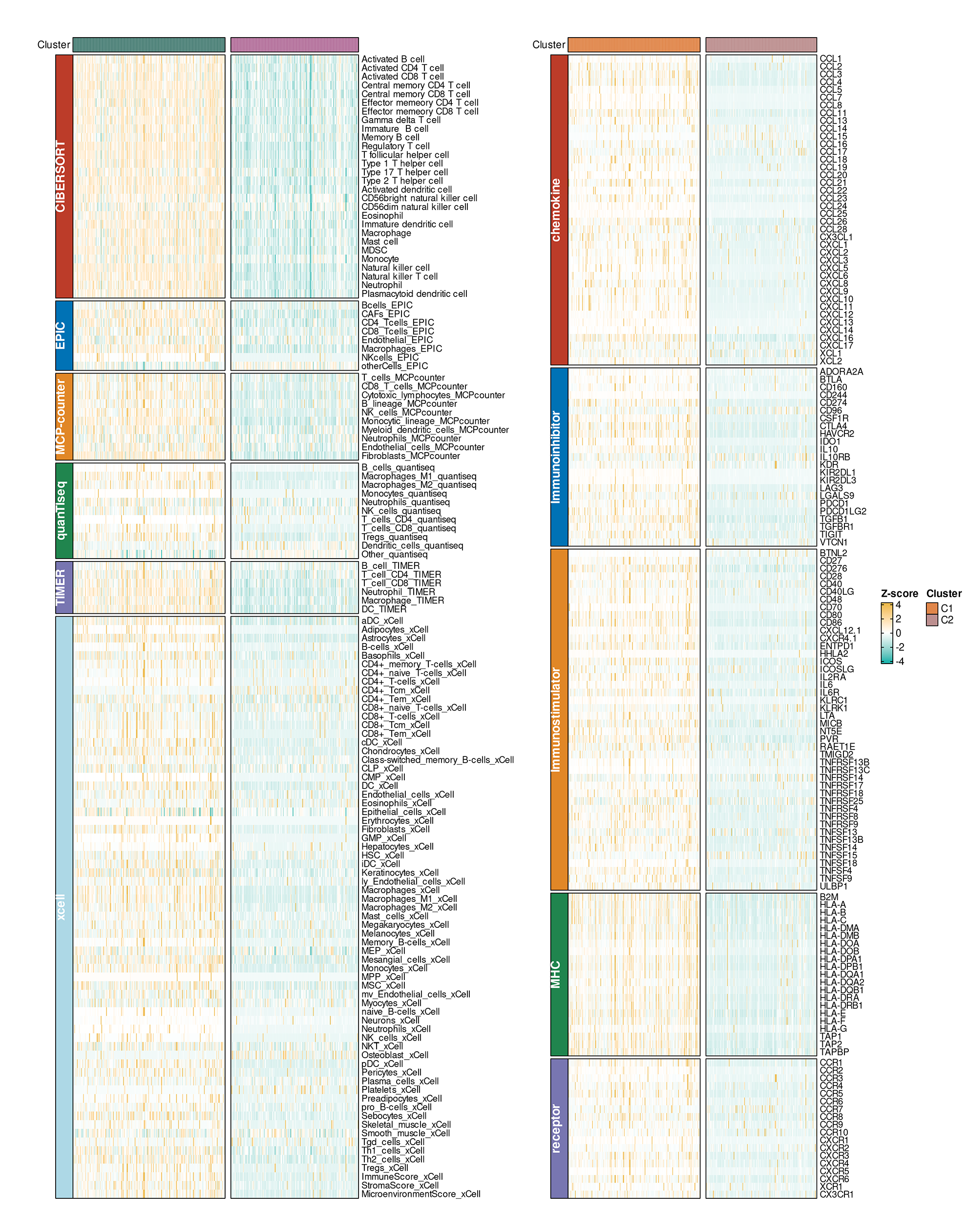

Supplement: Supplementary file 1 [file ijms-24-04760-s001.zip › Figure S3.tif]

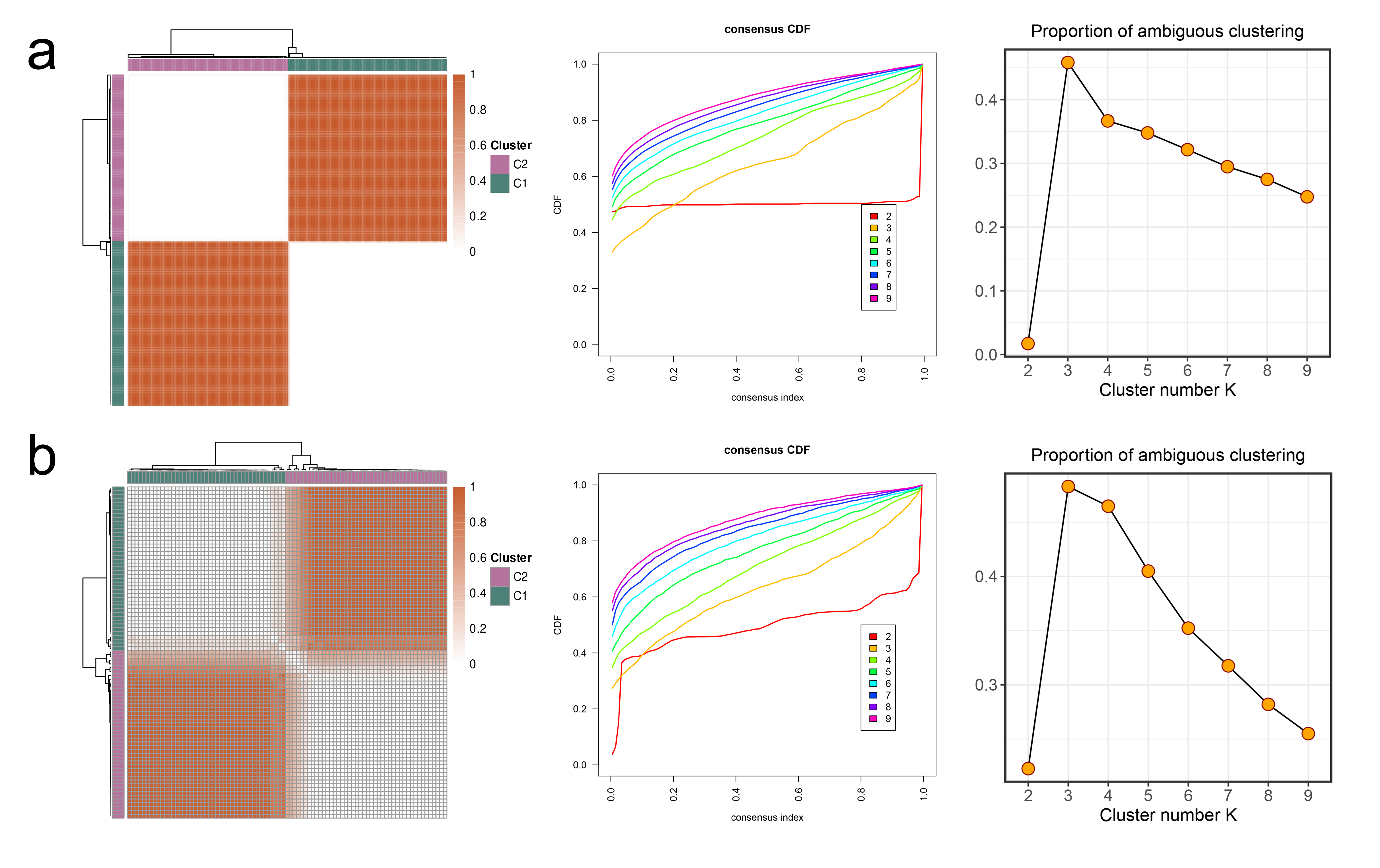

Supplement: Supplementary file 1 [file ijms-24-04760-s001.zip › Figure S4.tif]
